# Phenotypic Detection Methods, Clinical Outcomes, and Therapeutic Strategies for OXA-Type Carbapenemase-Producing Acinetobacter Species in an Indian Tertiary Care Setting

**DOI:** 10.7759/cureus.109683

**Published:** 2026-05-26

**Authors:** Pranay R Ullengala, Ravindra V Shinde, Satish R Patil

**Affiliations:** 1 Department of Microbiology, Krishna Institute of Medical Sciences, Krishna Vishwa Vidyapeeth (Deemed to be University), Karad, IND

**Keywords:** acinetobacter baumannii, acinetobacter species, carbapenemases, esbl, multidrug resistance (mdr), oxa-type carbapenemases (chdls), sulbactam-durlobactam (sul-dur)

## Abstract

*Acinetobacter *species have emerged as significant nosocomial pathogens, severely complicating treatment protocols in tertiary care settings due to their rapid development of multidrug resistance (MDR). As an opportunistic pathogen, *Acinetobacter baumannii* is frequently associated with ventilator-associated pneumonia (VAP) and bloodstream infections, carrying substantial mortality rates. The primary mechanism driving this extensive beta-lactam resistance and the focal point of this review is the production of OXA-type carbapenem-hydrolyzing class D β-lactamases (CHDLs), alongside extended-spectrum beta-lactamases (ESBLs). The pathophysiology, virulence factors, clinical symptoms, and epidemiology of these resistant bacteria are all reviewed in this thorough analysis. Further, it describes the essential laboratory challenges and standard phenotypic methodologies needed to precisely isolate and profile these highly resistant *Acinetobacter* species from clinical specimens, as well as the molecular mechanisms underlying carbapenem resistance. Finally, given the high prevalence of these isolates in Indian intensive care units, this review evaluates the severe clinical outcomes associated with these strains and highlights essential therapeutic strategies. It emphasizes the urgent need for tailored combination therapies and explores the efficacy of novel targeted treatments, such as the sulbactam-durlobactam combination, in managing severe OXA-driven infections.

## Introduction and background

The genus* Acinetobacter* is characterised by gram-negative, strictly aerobic, non-fermenting, non-fastidious, non-motile, and catalase-positive, oxidase-negative coccobacillary bacterium [1]. *A. baumannii, A. calcoaceticus*, and 12 other DNA groups or genospecies were identified by the standard classification of *Acinetobacter*, which was based on DNA-DNA hybridization experiments [2]. *A. lwoffii, A. haemolyticus, A. johnsonii, and A. junii* are also frequently isolated in clinical settings [3]. *Acinetobacter* spp. causes various conditions, including pneumonia, septicemia, wound sepsis, urinary tract infections, endocarditis, meningitis, and bloodstream infections (BSI) [4].

*A. baumannii *isolates have been identified with significant multidrug resistance (MDR), typically described as being resistant to at least three different classes of antibiotics [5]. Furthermore, many clinical isolates exhibit extensive resistance due to extended-spectrum beta-lactamases (ESBLs) and metallo-β-lactamases (MBLs) gene transcription, making the treatment of *Acinetobacter *infections increasingly challenging [6,7]. The global dissemination of these resistant clones is driven by complex epidemiological factors and diverse molecular mechanisms, requiring a thorough understanding of their transmission and therapeutic management [8-10]. Recent advancements in laboratory diagnostics and the development of novel agents are essential to address the rising mortality rates associated with these infections [11-13]. Consequently, evaluating the efficacy of emerging treatments like sulbactam-durlobactam SUL-DUR is critical for clinical success [14].

## Review

Methods

Research Strategy

A comprehensive search strategy was developed to identify relevant literature on *A. baumannii* and OXA-type carbapenemase production in tertiary care settings. The search was structured using key concepts, including the target organisms (*A. baumannii* or *Acinetobacter *spp.), and resistance mechanisms (OXA-type β-lactamases, carbapenem-hydrolyzing class D β-lactamases (CHDLs), carbapenem resistance, blaOXA genes), detection methods (phenotypic tests such as CarbAcineto NP, modified Hodge test, and advanced methods like PCR and matrix-assisted laser desorption/ionization time-of-flight (MALDI-TOF)), clinical outcomes (mortality, ventilator-associated pneumonia, bloodstream infections), and therapeutic strategies (colistin, tigecycline, SUL-DUR, combination therapy). Boolean operators (AND, OR) were used to combine these keywords into structured search strings to maximize retrieval of relevant studies.

Electronic databases, including PubMed/MEDLINE, Scopus, Web of Science, and Google Scholar, were systematically searched for studies published between 2010 and 2025. Inclusion criteria comprised peer-reviewed articles focusing on clinical, microbiological, and epidemiological aspects of OXA-producing* Acinetobacter *infections in humans, while non-clinical studies, articles without specific reference to OXA-type enzymes, and non-English publications were excluded. Filters such as publication year, study type, and human subjects were applied to refine the search. This strategy ensured a focused and comprehensive collection of evidence to support the review of phenotypic detection methods, clinical outcomes, and treatment approaches

**Figure 1:**
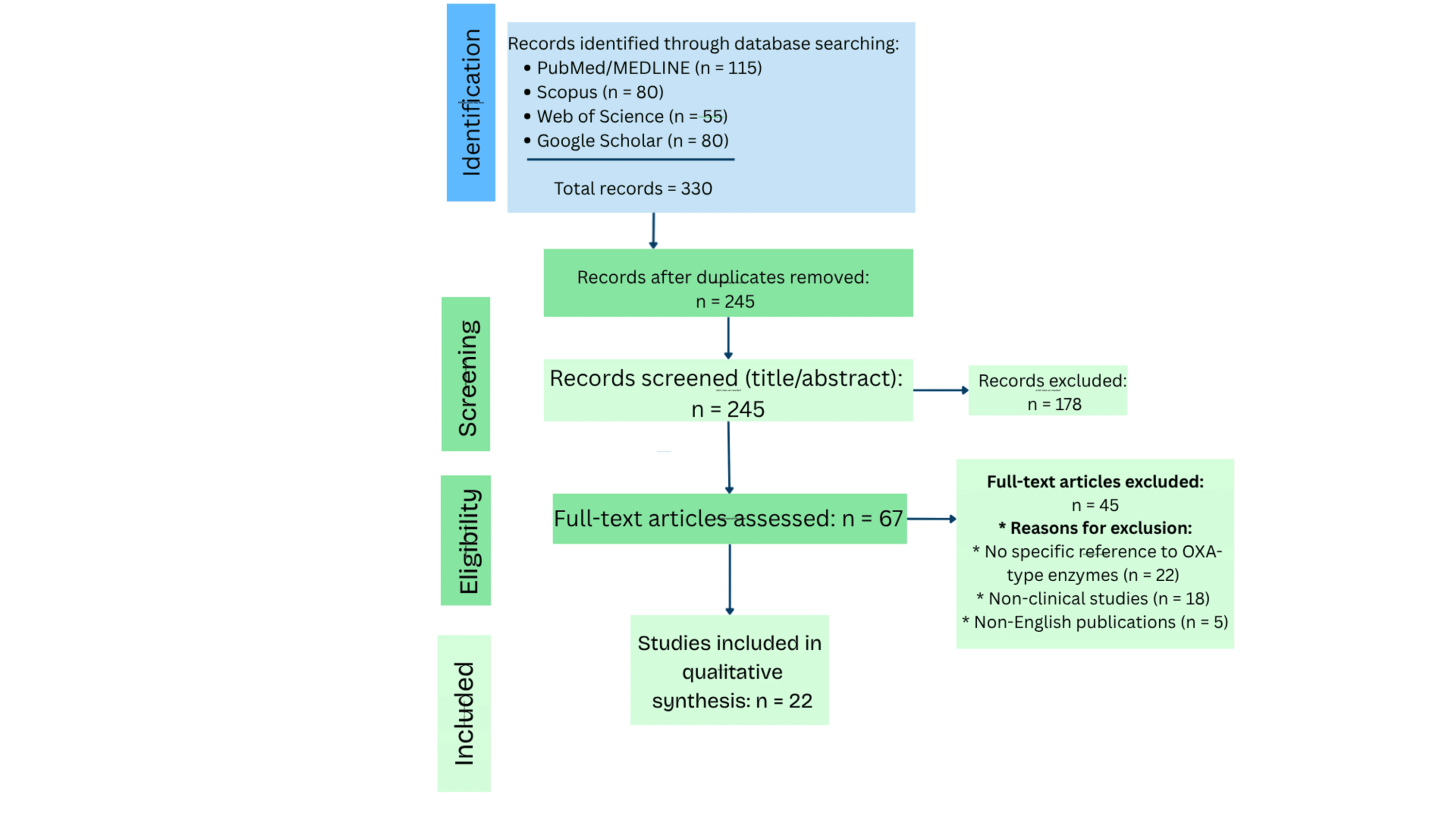
Flow diagram of study selection process.

Studies that assessed phenotypic detection methodologies, clinical outcomes, and therapeutic strategies, and focused on OXA-type carbapenemase-producing *Acinetobacter* species, were considered. Excluded were studies that were non-clinical in nature, lacked specific reference to OXA-type enzymes, and were not published in English. To ensure clinical relevance, focus was placed on established resistance mechanisms, epidemiology, and targeted treatment protocols. This review is a narrative synthesis. We did not calculate pooled effect sizes or statistical significance. Instead, findings were qualitatively synthesized based on the included studies (Figure 1).

Pathogenesis and virulence factors

**Figure 2:**
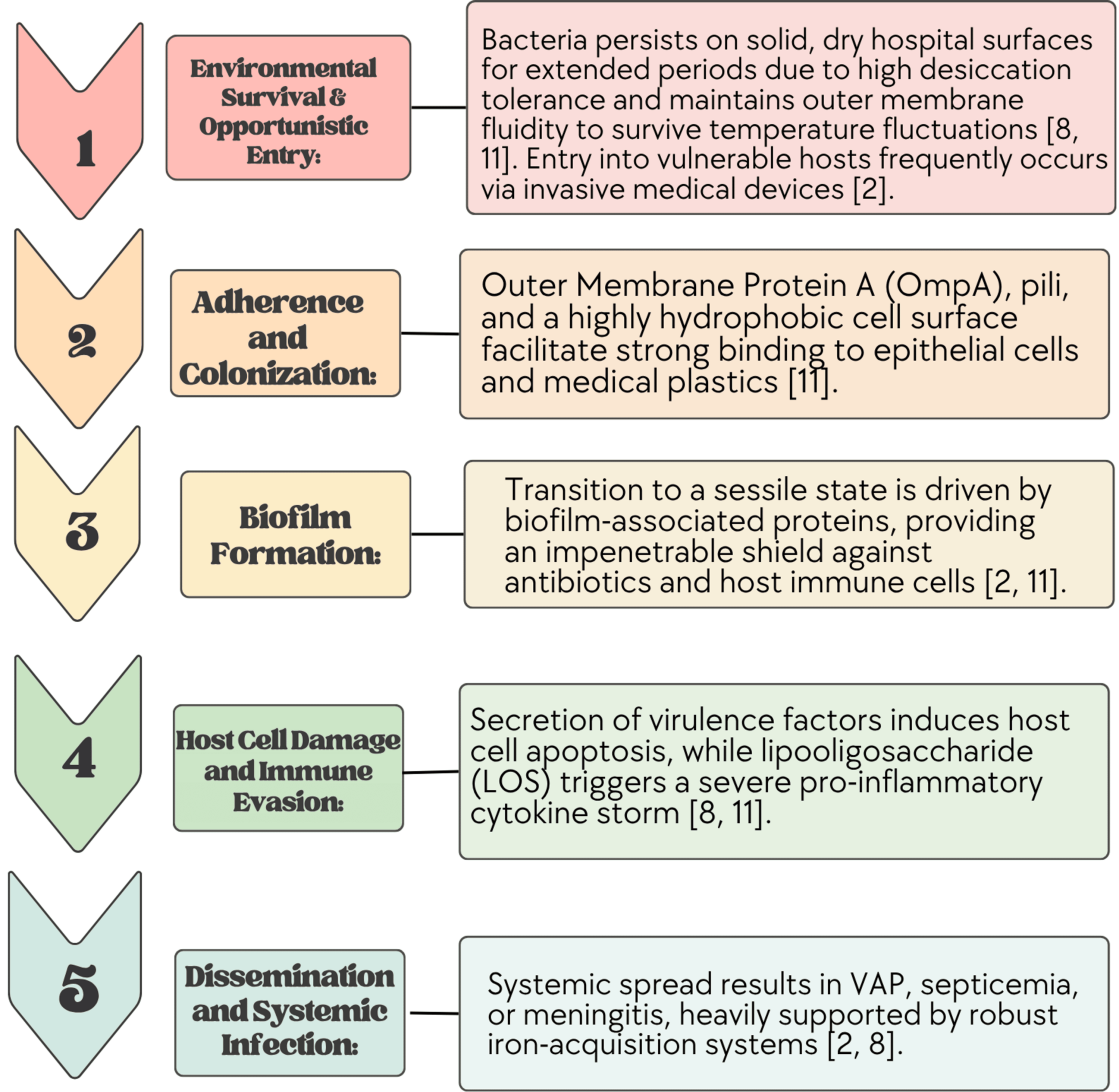
The Infection Cycle. Key stages include environmental survival, adherence, biofilm formation, host cell damage, and systemic dissemination [2,8,11]. Abbreviations: LOS: Lipooligosaccharide; OmpA: Outer Membrane Protein A; VAP: Ventilator-Associated Pneumonia.

The pathogenicity of *Acinetobacter* relies heavily on its versatile genetic machinery and genomic plasticity, allowing rapid adaptation to environmental stress and host immune responses [1,2]. The infection cycle and key virulence mechanisms are summarized in Figure 2 [2,8,11].

Historical emergence of OXA-type carbapenemases

The clinical significance of* Acinetobacter baumannii* has been dramatically shaped by its acquisition and expression of Ambler Class D beta-lactamases, commonly known as oxacillinases (OXA) [3,4].

A clinical strain of *A. baumannii *that was obtained in 1985 at the Royal Infirmary of Edinburgh, Scotland, had the first carbapenem-hydrolyzing OXA enzyme [3]. Additionally, this isolate was obtained prior to the widespread use of imipenem in the clinical practice of that institution [3]. The encoding gene was first identified as ARI-1 (*Acinetobacter *Resistant to Imipenem), however it was later sequenced in 1993 and formally defined as OXA-23 [3,4].

Functional Profiles and Substrate Specificities of OXA Subgroups

During the late 1990s and early 2000s, several subgroups of acquired OXA carbapenemases appeared worldwide after OXA-23 was characterised [4]. The OXA-58 group was found in France in 2003, whereas the OXA-24/40 group was initially detected in a highly resistant strain from Spain in 1997 [3,4]. Concurrently, researchers identified the OXA-51-like family, which, unlike the acquired enzymes, was found to be intrinsic and chromosomally mediated in naturally occurring *A. baumannii *strains [4].

Although all OXA-type CHDLs possess the capability to hydrolyze carbapenems, their origins, functional capacities, and substrate specificities vary significantly [3,4]. Intrinsic OXA-51-like enzymes generally exhibit minimal baseline carbapenemase activity, conferring clinical resistance primarily when significantly overexpressed through the upstream insertion of potent promoters such as ISAba1 [4,7]. In contrast, the acquired subgroups, primarily OXA-23, OXA-24/40, and OXA-58, have strong carbapenem hydrolyzing activity and are extremely mobile, spreading quickly via plasmids and transposons [7,9].

Regarding substrate profiles, these enzymes efficiently hydrolyze penicillins (e.g., ampicillin, ticarcillin, piperacillin) and carbapenems (e.g., imipenem, meropenem) [3,7].

However, they generally display weak hydrolytic activity against extended-spectrum cephalosporins [7]. Crucially, OXA enzymes are notably resistant to classic β-lactamase inhibitors such as clavulanic acid and tazobactam [7]. This intrinsic inhibitor resistance highlights the therapeutic necessity of novel diazabicyclooctane inhibitors, such as durlobactam, which are specifically designed to neutralize these Class D enzymes [14].

Genetic Mobilisation and the Function of Transposons

Although *A. baumannii* possesses intrinsic, low-level OXA genes, the true clinical crisis began when these bacteria started exchanging acquired OXA genes through horizontal gene transfer [4,5]. 

The rapid development of these resistance mechanisms is predominantly driven by highly mobile genetic elements such as conjugative plasmids and transposons [5]. For example, the interaction of the *bla*OXA-23 gene with insertion sequences, specifically ISA*ba1*, contributes significantly to its rapid proliferation. In addition to allowing for easy genetic mobility across plasmids, which allows resistance to spread exponentially from one bacterium to another, this insertion sequence also acts as a powerful promoter, dramatically increasing the production of the OXA enzyme itself [4,5].

Transition to Global Endemicity

By the mid-2000s, OXA-producing clones evolved from localized European incidents to a worldwide threat [4]. Carbapenem-resistant *A. baumannii* (CRAB) infections are still mostly caused by the OXA-23 cluster, which maintains the pathogen's position as a major public health emergency [5].

The rapid global dissemination of these OXA-producing* A. baumannii* clones is not solely driven by intrinsic bacterial factors but is heavily facilitated by modern human activities [8]. Key drivers include international medical tourism, as patients seeking care across borders inadvertently transport and introduce resistant strains into new healthcare facilities [8]. Furthermore, cross-border transfer of critically ill patients, international travel, and mass migration events have all served as primary conduits, transforming localized outbreaks into the widespread, multinational endemicity we see today [8].

Molecular mechanisms of carbapenem resistance: the role of OXA and CHDLs

ESBLs produce resistance to broad-spectrum cephalosporins, whereas carbapenem resistance in *Acinetobacter *is predominantly facilitated by Ambler Class D CHDLs, often known as oxacillinases [1, 6]. These enzymes are divided into intrinsic and acquired categories. Regarding intrinsic CHDLs, a chromosomally expressed blaOXA-51-like gene is present in the majority of* A. baumannii* isolates. When an addition sequence, such as ISAba1, inserts upstream and increases the activity of the enzyme, resistance develops [6,7]. Conversely, the global spread of acquired CHDLs in CRAB is mostly due to horizontal gene transfer through transposons and plasmids, with OXA-23, OXA-24/40, and OXA-58 being the most common clusters [7-9]. Additionally, the NDM, VIM, and IMP types of Ambler Class B MBLs are extremely powerful carbapenemases that significantly contribute to resistance profiles in clinical isolates [6,9].

Other mechanisms of drug resistance

While the main cause of resistance is the synthesis of β-lactamases (such as OXA-type CHDLs and MBLs),* A. baumannii* uses a number of additional innate and acquired mechanisms. Among these include the overexpression of multidrug efflux pumps, such as the AdeABC, AdeFGH, and AdeIJK systems, which actively extrude a wide range of antibiotics including tigecycline and fluoroquinolones [7,10].

Furthermore, the loss or down-regulation of outer membrane porins (such as CarO and Omp33-36) significantly decreases membrane permeability, preventing drug entry [10]. Alterations in target sites, such as mutations in penicillin-binding proteins (PBPs) or DNA gyrase, further compound the MDR profile of this pathogen [7].

Intrinsic Resistance Profile

In addition to acquired mechanisms, *A. baumannii *has an extremely high baseline of intrinsic resistance. This natural defense is primarily mediated by chromosomal expression of AmpC-type cephalosporinases (ADCs), which degrade aminopenicillins and early-generation cephalosporins [7]. Furthermore, the bacterium's outer membrane permeability is significantly lower than that of many other gram-negative pathogens, forming a natural, highly restrictive physical barrier against a wide range of bulky antimicrobial drugs [10].

Clinical manifestations and OXA-mediated infection outcomes

Many life-threatening illnesses are caused by *Acinetobacter* spp., which mostly affect critically ill patients and immunocompromised people [9,10].

The most severe manifestations of respiratory and bloodstream infections include hospital-acquired pneumonia (HAP) and ventilator-associated pneumonia (VAP). VAP is particularly prevalent in intensive care units (ICUs) because *A. baumannii* exhibits a strong propensity to adhere to endotracheal tubes and form resilient biofilms, making eradication exceptionally difficult once colonization occurs [2,10]. These severe respiratory infections frequently progress to secondary septicemia, which is significantly exacerbated by prolonged mechanical ventilation [10].

Other infections, such as endocarditis, surgical site infections, wound sepsis, and urinary tract infections, are also common in tertiary settings [11,12].

Specific Outcomes of OXA-CHDL Infections

In tertiary care, the clinical effect of CRAB, which is mostly caused by OXA-type enzymes like OXA-23, is particularly severe [13,14]. OXA-23-producing strains have extremely high fatality rates and have been linked to severe outbreaks in critical care units [15-17].

Infections caused by OXA-producing strains can result in 30-day mortality rates exceeding 71%, particularly when the initial antimicrobial therapy administered is inappropriate [13]. Patients infected with OXA-23 generating clones have significantly worse clinical outcomes than those with susceptible strains, with mortality being directly associated with the resistance profile. At-risk populations include solid organ transplant recipients, such as liver transplant patients, those receiving hemodialysis treatment, and critically sick patients required extended mechanical ventilation or invasive nasogastric tubes, who are most susceptible to contracting OXA-producing CRAB [16,17].

Severe thrombocytopenia, the need for mechanical ventilation, and crucial delays in the administration of active, suitable antibiotics are important symptoms of mortality in patients infected with OXA-mediated multidrug-resistant strains [13].

Epidemiology

A public health emergency is the spread of multidrug-resistant *A. baumannii *over the world [8,9]. CRAB, which has spread around the world to become endemic in South and Southeast Asia, has been classified by the WHO as a serious, top-priority disease [8,16]. 

**Figure 3:**
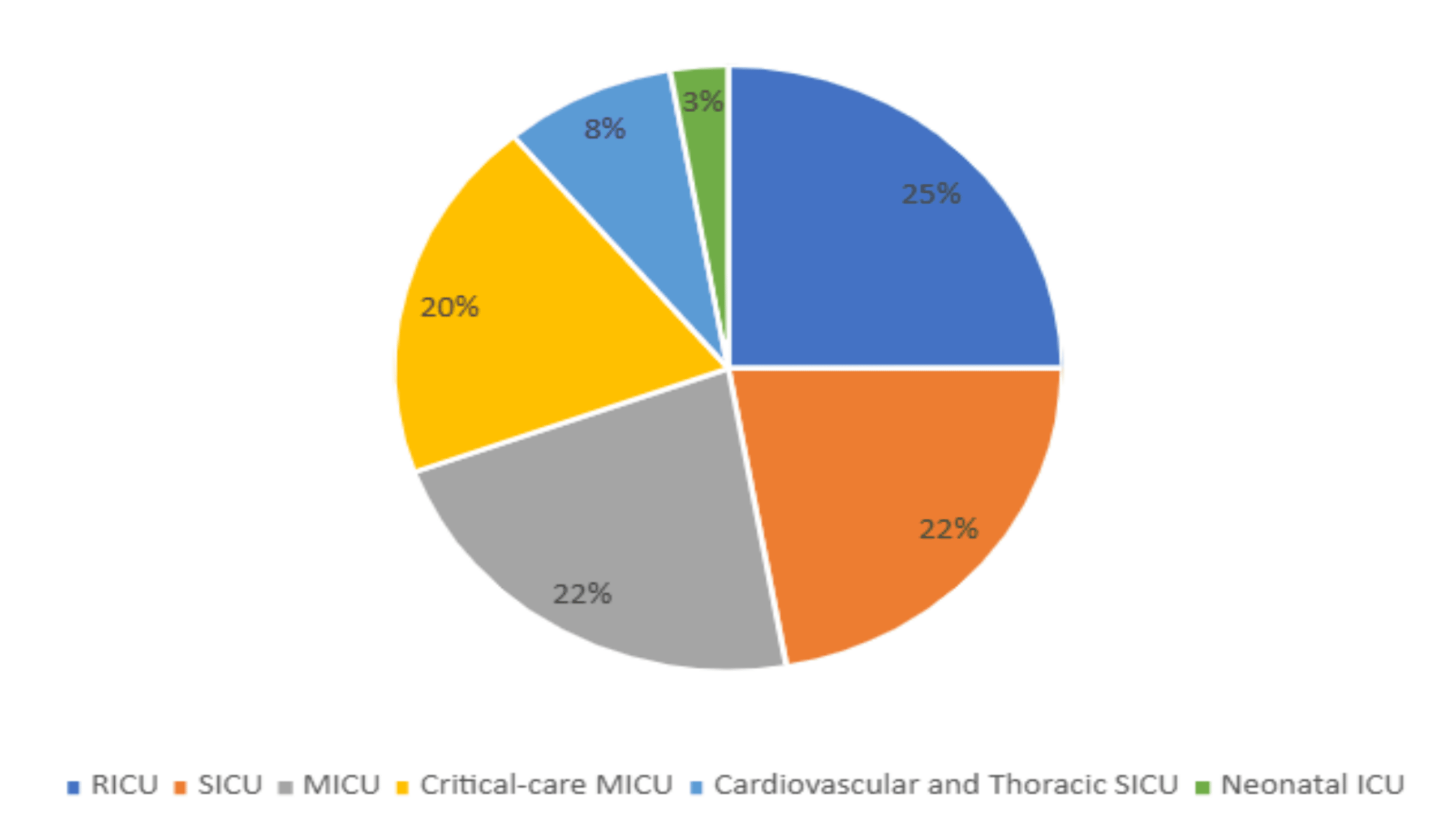
Distribution of MBL-producing XDR isolates across Intensive Care Units. Percentage distribution of resistant bacterial isolates across different clinical environments [21,22]. Abbreviations: MBL: Metallo-β-Lactamase; XDR: Extensively Drug-Resistant; RICU: Respiratory Intensive Care Unit; SICU: Surgical Intensive Care Unit; MICU: Medical Intensive Care Unit.

*Acinetobacter* species infections are extremely common in tertiary care institutions in India. Prevalence rates in ICU settings are consistently above 20%, according to recent surveillance data from many states, including Haryana [11], Tamil Nadu [15,18], Gujarat [12,19], and Rajasthan [20]. The majority of carbapenem-resistant ICU isolates in Maharashtra, in particular, have the blaOXA-23 gene, frequently co-exist with metallo-beta-lactamases, and exhibit strong biofilm formation, according to recent audits from tertiary care facilities. The distribution of MBL-producing extensively drug-resistant (XDR) isolates across ICUs is shown in Figure 3 [21,22].

Laboratory challenges in detection

Significant clinical laboratory challenges exist for the accurate phenotypic identification and detection of ESBL and carbapenemase producers [6,21].

Taxonomic complexity presents a major hurdle, as it is challenging to distinguish closely related species within the *A. calcoaceticus-A. baumannii* (ACB) complex - mainly *A. baumannii*,* A. calcoaceticus*,* A. pittii,* and *A. nosocomialis* - using standard phenotypic features, frequently required complex genotypic techniques [7,10].

Furthermore, detection sensitivity is compromised because standard beta-lactamase inhibitors have little effect on CHDLs (Class D enzymes), which have minimal intrinsic hydrolytic activity against carbapenems. As a result, when screening OXA producers, conventional synergy tests often provide false-negative findings [6,7,21].

Detection of OXA-type carbapenemases (CHDLs) and available tests

Specialised diagnostic techniques are necessary since traditional synergy tests intended for MBLs or Class A carbapenemases can generate false-negative findings when screening OXA producers [6,21]. There are currently five main test categories that are used to identify OXA CHDLs.

Biochemical and colorimetric assays, such as the updated CarbAcineto NP test, offer improved sensitivity and can be used to quickly identify carbapenemases specifically among *Acinetobacter baumannii* [21]. 

Rapid immunochromatographic lateral flow tests that allow for the direct identification of certain acquired OXA enzymes can be directly identified from positive blood cultures or bacterial colonies using targeted antibody-based tests [7]. 

Molecular diagnostics and genotypic assays, including multiplex PCR and whole-genome sequencing (WGS), continue to be the gold standard for identifying blaOXA genes and resolving taxonomic complexity due to phenotypic unreliability [2,7]

MALDI-TOF mass spectrometry is a cutting-edge method that measures the mass spectra of imipenem breakdown products to assess carbapenemase activity [7].

Phenotypic growth-based screening, such as the modified Hodge test (MHT), utilizes a cloverleaf deformation in the growth of a susceptible indicator organism surrounding a carbapenem disc to screen for carbapenemase synthesis [6].

Standard laboratory methodology

To systematically isolate and profile *Acinetobacter* species, a rigorous phenotypic approach is implemented. Clinical specimens are initially cultured on MacConkey and blood agar [11,15]. Suspect colonies revealing gram-negative coccobacilli undergo the necessary biochemical tests to confirm their identity [12]. Following identification, antibiotic resistance patterns are assessed using the Kirby-Bauer disk diffusion method on Mueller-Hinton agar (MHA) [11,15,19]. Phenotypic detection of carbapenemases is primarily screened using the MHT or assessed via the imipenem-EDTA double disc synergy test (DDST) to isolate MBL production [6,20]. Similarly, phenotypic detection of ESBLs is achieved using the DDST by measuring inhibition zones between cephalosporin discs with and without clavulanic acid [1,20].

Automated Identification and Susceptibility Testing Systems

In modern tertiary care settings, manual phenotypic methods are increasingly supplemented or replaced by automated platforms that offer faster turnaround times and standardized results. Some commonly used systems include the VITEK 2 (bioMérieux, Marcy-l'Étoile, France), BD Phoenix (Becton Dickinson, Franklin Lakes, NJ, USA), and MicroScan WalkAway (Beckman Coulter, Brea, CA, USA). These platforms use colorimetric or fluorometric technologies to quickly identify species and determine minimum inhibitory concentrations (MICs) for a wide range of antimicrobials, which is critical for managing MDR *Acinetobacter* infections [10,11].

Therapeutic strategies and effective antimicrobial agents

OXA-producing CRAB are extremely difficult to treat since they often show MDR and are resistant to nearly all beta-lactams [1,7]. Antibiotic regimens that are carefully customised are necessary for management.

Historically, salvage therapy for severe CRAB infections has depended heavily on polymyxins, such as colistin and polymyxin B. However, nephrotoxicity restricts their usage, and the development of colistin and fosfomycin resistance in ICUs is a growing clinical concern [1]. 

Sulbactam has intrinsic bactericidal activity against *Acinetobacter*, and durlobactam is a novel, broad-spectrum diazabicyclooctane beta-lactamase inhibitor that effectively protects sulbactam from degradation by Ambler Class D (OXA) carbapenemases [14]. SUL-DUR is a highly effective, novel targeted therapy specifically designed for CRAB. Even in extremely sensitive groups like liver transplant recipients, SUL-DUR has shown remarkable effectiveness in treating serious infections, including OXA-23-driven pneumonia [14]. 

Tetracycline derivatives, specifically minocycline and high-dose tigecycline, are commonly used as part of combination regimens because of their prolonged in vitro efficacy against several MDR *Acinetobacter *bacteria [13].

More recently, eravacycline, a novel synthetic fluorocycline, has emerged as a potent alternative. It was developed to evade common tetracycline-specific resistance mechanisms, including ribosome protection and particular efflux pumps. Clinical evidence suggests that eravacycline has significant in vitro activity against CRAB isolates and could be a beneficial component of combination regimens for complex intra-abdominal and respiratory infections [13,22].

To achieve a clinical cure and overcome resistance mechanisms, prompt administration of combination therapies (such as a polymyxin combined with tigecycline, fosfomycin, or a high-dose carbapenem) is frequently necessary due to the high mortality predictors associated with OXA-CHDL infections [1,13].

The Role of Cefiderocol

Cefiderocol represents a significant advancement in treating CRAB. As a novel siderophore cephalosporin, it utilizes the bacterium’s own iron-transport systems to bypass the outer membrane barrier, effectively acting as a "Trojan Horse" to deliver the drug directly to its target. Cefiderocol is stable against a variety of beta-lactamases, including OXA-type CHDLs and MBLs, making it an important treatment choice for XDR infections [14,21].

## Conclusions

*Acinetobacter *species, especially *Acinetobacter baumannii*, pose a serious threat in tertiary care settings because of their quick development of MDR. This opportunistic bacterium is the main cause of serious nosocomial infections, including bloodstream infections and VAP, which have high death rates. Clinical care is particularly challenging because of this bacterial ability to persist on hospital surfaces, create impenetrable biofilms, and cause severe human immune responses. The pathogen resistance mechanisms, which are primarily caused by the production of ESBLs and Ambler Class D OXA, significantly complicate the treatment landscape. OXA-type enzyme-mediated infections, especially OXA-23, are linked to extremely high 30-day death rates that can reach 71%. Furthermore, it is extremely difficult to precisely detect these resistance profiles in the lab. When searching for OXA producers, traditional phenotypic synergy testing sometimes provides false-negative findings. Rapid diagnostic instruments like the CarbAcineto NP test are essential for the prompt and accurate detection of carbapenemases, even if molecular genotypic tests continue to be the gold standard for resolving taxonomic complexity. As resistance to traditional recovery therapies like polymyxins continues to emerge, establishing effective treatment protocols is more urgent than ever. Dealing with these XDR isolates usually requires a prompt, tailored combination of therapies to achieve a clinical cure. Additionally, even among extremely sensitive groups of patients, the introduction of breakthrough, targeted agents - like the SUL-DUR combination - offers a very promising and successful treatment strategy for controlling severe, OXA-driven* Acinetobacter* infections. 
